# An uncommon presentation of an VIII nerve tumor

**DOI:** 10.1016/S1808-8694(15)30615-7

**Published:** 2015-10-18

**Authors:** Rubem Cruz Swensson, Rogério Poli Swensson, Fabio Eduardo Caramante Pizzini, Pedro Robson Boldorini, José Jarjura Jorge Júnior

**Affiliations:** 1 ENT, assistant in the discipline of Otorhinolaryngology at Faculdade de Medicina de Sorocaba da PUC-SP.; 2 ENT resident at Faculdade de Medicina de Sorocaba da PUC-SP.; 3 ENT resident at Faculdade de Medicina de Sorocaba da PUC-SP.; 4 ENT, volunteer physician in the discipline of Otorhinolaryngology at Faculdade de Medicina de Sorocaba da PUC-SP.; 5 PhD in ENT, Professor at the Department of Surgery and Otorhinolaryngology Coordinator at Faculdade de Medicina da PUC-SP. Pontifícia Universidade Católica de São Paulo Faculdade de Medicina de Sorocaba.

**Keywords:** deafness, schwannoma, viii nerve tumor

## Abstract

Vestibular Shwannomas are responsible for 80-90% of the cerebelar-pontine angle tumors and their incidence is of 0.8 to 20.5% of all tumors. Unilateral and progressive hearing loss is the most frequent and premature symptom, and tinnitus is the second most common complaint. Only 5% of the patients have normal audiograms. In this case the patient complained of ipsilateral facial numbness and weak blink, posterior pinna hypoesthesia (Hitzelberger +), tear reduction and positive Romberg test. He also had mouth twisting but no other involvement of other cranial nerves. Hearing acuity was normal.

## INTRODUCTION

Pontocerebellar angle tumors in their various manifestations occur quite frequently in the ENT practice. They account for 2-10% of all intracranial tumors^1^, ^2^, ^3^, and may be lethal if not properly treated. Vestibular schwannomas (VS) amount to 80-90% of all pontocerebellar angle tumors^1^, ^4^, ^5^. They are slow-growth, benign tumors that originate in the vestibular segment of the VIII cranial nerve, mostly in the upper division of this segment^1^, ^3^, ^6^, ^9^. These tumors derive histologically from Schwann cells, and appear more commonly inside the internal auditory meatus^1^, ^2^, ^3^, on the transition between central and peripheral myelin, known as the Obsteiner-Redlich zone.

Malignant tumors in the VIII cranial nerve are extremely rare^1^.

## LITERATURE REVIEW

Vestibular schwannomas were first described by Sandifort in 1777, while Cushing, in 1917, described the natural history of the tumor, suggesting bilateral suboccipital craniotomy with subtotal removal of the tumor as the treatment of choice.

The current prevalence rate of vestibular schwannomas is not accurately known. It is estimated to range between 0.8% and 2.5%^1^, ^2^, ^6^, ^7^. Patients of all ages have been diagnosed with VS, but the tumor is more frequently found in people over the age of fifty^1^, ^2^, ^8^, ^9^. Women are more involved^1^, ^2^, ^9^, ^10^, in a ratio close to 3:2 in relation to males^9^. Ethnicity appears not to be significant^1^.

Macroscopically, the tumor is encapsulated and has a smooth surface^1^, ^2^, ^3^.

From the standpoint of histopathology, vestibular schwannomas can be classified in two varieties. The first and most common^1^, ^11^ is Antoni's type A, characterized by the prevalence of argyrophil fibers and nuclei arranged in palisades that form Verocay bodies when arranged circularly^1^, ^2^, ^9^, ^11^. Antoni's type B is found in larger tumors^1^ and is characterized by round, picnotic nuclei with various cystic and microcystic formations^1^, ^2^, ^9^.

VS may occur in two fashions: sporadic and associated with type-2 neurofibromatosis (NF - 2). In the first, the tumor is unilateral and accounts for about 95% of the cases. When associated with NF - 2, the tumor is typically bilateral and accounts for 5% of all cases^1^, ^2^, ^6^, ^8^, ^9^.

Progressive unilateral hypoacusis is the earliest and most frequent symptom, involving 75-95% of the patients. Sudden deafness occurs in 8-26% of the cases^1^, ^2^, ^3^, ^5^, ^6^, ^8^, ^9^, ^12^, ^13^, although some studies have found prevalence rates greater than 26%^2^. On the other hand, vestibular schwannomas are the etiology for sudden deafness in only 1-2% of the patients. Aural fullness may also occur in association with fluctuating hypoacusis, simulating Ménière's syndrome^1^, ^3^, ^9^. Approximately 5% of the patients diagnosed with vestibular schwannomas have normal hearing^3^, ^13^, ^14^.

Tinnitus is the second most frequent complaint, appearing in as much as 60-86% of patients^1^, ^9^, both as an isolated symptom or in conjunction with deafness. Continuous or fluctuating tinnitus, associated with the unilateral manifestation of the symptom, is a warning sign to include vestibular schwannoma in the diagnostic possibilities^1^, ^2^, ^6^, ^8^, ^9^.

Slow tumor growth and consequent vestibular adaptation and compensation render vertigo-like symptoms uncommon. When present, such symptoms occur early on and tend to disappear as the disease develops^1^, ^2^, ^3^, ^6^, ^9^. In a recent study conducted by Selesnick et al., the symptom was present in 19% of the patients^2^, ^15^. An increasing feeling of instability and unbalance secondary to tumor growth is a relatively common symptom^1^, ^2^, ^3^, ^6^, ^9^, probably the result of cerebellar compression^1^, ^9^.

Hypoesthesia and facial pain may occur when the tumor is large enough to compress the V cranial nerve. The corneal reflex may be impaired in such cases^1^, ^2^, ^3^,9. Facial paralysis is observed in rare cases of patients with very large tumors^1^, ^2^, ^9^.

The patient's static and dynamic balance may be altered, depending on the size of the tumor^1^, ^15^. Nystagmus is occasionally found, usually when vertigo-like symptoms are present^1^, ^9^. Clinical examination of all other cranial nerves is imperative, mainly of the V, VI, VII, XIX, X, XI, and XII nerves^1^, ^9^.

Tonal audiometry classically shows unilateral sensorineural hearing loss, without the typical curve pattern obtained under tympanometry^1^, ^2^, ^3^, ^9^, ^13^. Studies have shown that only 5% of the patients with vestibular schwannoma have normal audiometric test results^3^, ^12^. Voice discrimination is also usually altered and roll-over is present^1^, ^2^, ^3^, ^9^.

The most characteristic finding of electronystagmography is labyrinthine hyporeflexia or areflexia under caloric tests in the involved side^1^, ^2^, ^9^.

Brainstem evoked response audiometry (BERA), a more sensitive and specific test used to detect alterations introduced by tumors, has sensitivity levels of 93-98%^1^, ^2^, ^3^, ^12^, ^9^. The most important findings are increased interval between waves I and III above 2.3 ms; increased interval between waves I and V greater than 4.4ms; interaural time difference on wave V greater than 0.4 ms, and absence of wave I^1^, ^2^.

Other tests such as electrocochleography may be performed when wave I cannot be seen in the BERA, and electroneuromyography of the facial nerve to aid in the diagnosis of vestibular schwannoma1.

Temporal bone CT and skull MRI scans are the diagnostic tools of choice for vestibular schwannomas, as their sensitivity levels are 95% and 100% respectively^1^, ^2^, ^3^, ^6^, ^9^.

Differential diagnosis is done against meningioma, Ménière's syndrome, tumors involving other cranial nerves (mainly the V cranial nerve), lipoma, hemangioma, and non-tumor lesions in the internal auditory meatus (vascular loops and neuritis)^1^, ^3^.

Observation, surgery and/or radiotherapy are the possible approaches to vestibular schwannoma^1^, ^2^, ^3^, ^8^, ^9^. The adoption of the first approach depends on the size of the tumor and on the clinical repercussions it brings about1. Surgery can be offered using the suprapetrous (via the middle fossa), retrosigmoid, and translabyrinthine approaches^1^, ^9^. Stereotactic radiosurgery was added to the therapeutic arsenal as a new option for patients in delicate clinical conditions and those who refuse to undergo surgery^1^.

## CASE STUDY

A. R. P., 37 years-old, male, born and residing in Votorantim, São Paulo, was admitted in the ENT ward of the Faculdade de Medicina da Pontifícia Universidade Católica de São Paulo (PUC - SP) at Sorocaba, complaining of physical instability during gait that had been evolving intermittently for the past two years. He had been treated at another center and given cinnarizine, and improved partially from the clinical symptoms. The patient claimed that his physical instability during gait worsened, with retropulsion combined with aural fullness in his right ear and ’wind-type’ ipsilateral tinnitus, sensation of paresthesia in the right side of the face; hypoacusis was not considered. The patient denied having high blood pressure, diabetes mellitus, and stated he did not smoke or drink alcohol. He had a left cornea transplant and no ophthalmic complaints.

**Figure 1:**
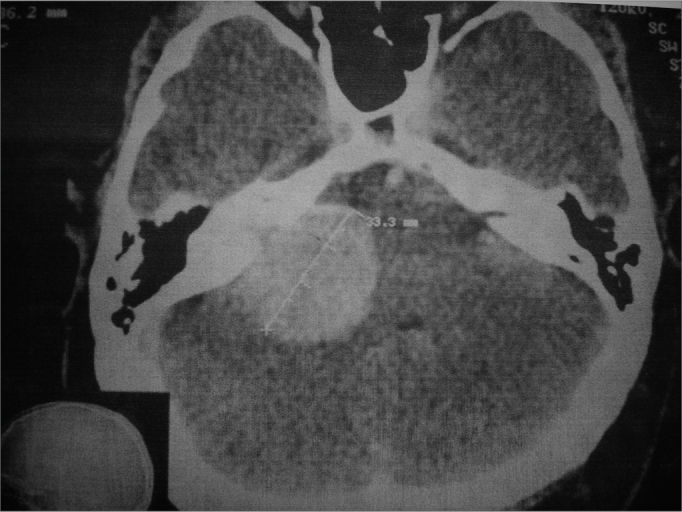
Mastoid CT scan showing a globus lesion (33.3mm in its largest axis), extending to the internal auditory meatus and enlarging it. We also noticed mass effect and compression of the cerebellum and pons.

Otoneurological and ENT examinations showed the following findings in the right dimer: right face hypoesthesia with reduced ipsilateral corneal reflex, hypoesthesia in the posterior-superior portion of the ear (positive for Hitselberg sign), reduced tearing, and positive sensitized Romberg. The labial sulcus was slightly deviated to the left, and no other alterations were observed in the other cranial nerves. Acoumetry did not identify changes to the hearing sensitivity of either of the airways, with a non-lateralized Weber and a positive Rinne bilaterally.

Audiological assessment did not point to any hearing impairment, as the patient's speech recognition was in perfect shape bilaterally. He had a C-type curve in tympanometry and presented stapedial reflexes. Areflexia was found in all otoneurological tests done on the patient's right ear. Metabolic exams showed presence of dyslipidemia.

A contrast enhanced CT scan of the ears was ordered to better assess the internal auditory meatus and the pontocerebellar angle. A globular tumor measuring 33.3mm in its largest dimension was found invading the right internal auditory meatus, where it grew larger and compressed the ipsilateral pontocerebellar transition zone (Picture 1).

The patient was referred to the Neurology and Neurosurgery service at Faculdade de Medicina da PUC SP to undergo BERA test and otoacoustic emissions tests. Dimenhydrinate was prescribed to deal with the patient's symptoms.

The patient never came back to the ENT ward at PUC-SP, as he is currently in custody and could not undergo the tests mentioned above. He is waiting to receive preoperative care at the Neurology and Neurosurgery service at Faculdade de Medicina da PUC - SP.

## DISCUSSION

Vestibular schwannomas amount to 80-90% of all pontocerebellar angle tumors^1^, ^4^, ^5^ and may be diagnosed in patients of all ages, although they are more commonly found in people over the age of fifty^1^, ^2^, ^8^, ^9^ and increased prevalence among women at a ratio of 3:^21^,^2^, ^9^, ^10^ when compared to males.

Even though progressive unilateral hypoacusis is the earliest and most frequent symptom, appearing in 75-95% of all patients, our patient was included in the 5% who have no hearing loss in spite of having vestibular schwannoma^3^, ^13^, ^14^. His speech recognition rate was 100%, a finding that goes against other papers in the literature, as voice discrimination is usually altered and affected by roll-over^1^, ^2^, ^3^, ^9^.

The patient complained of right unilateral tinnitus, which prompted us to look for a possible retrocochlear expansive process that would explain the unilateral humming associated with other vestibular symptoms^1^, ^2^, ^6^, ^8^, ^9^. According to the literature, tinnitus is the second most frequent complaint of vestibular schwannoma patients, as it is present in 60-86% of the cases^1^, ^9^.

The patient's main complaint was a growing feeling of instability and unbalance that resulted from the presence of a tumor in the pontocerebellar angle, a finding in perfect agreement with the literature, as approximately 70% of the tumors in this area measuring more than 30mm will produce such symptoms^15^.

Ear contrast enhanced CT with emphasis on the internal auditory meatus and pontocerebellar angle alongside skull MRI scans are the diagnostic tests of choice for vestibular schwannomas, as their sensitivity rates are 95% and 100% respectively^1^, ^2^, ^3^, ^6^, ^9^.

Involvement of the V and VII cranial nerves led to right face hypoesthesia, reduced ipsilateral corneal reflex, reduced tearing, hypoesthesia of the posterior-superior portion of the ear in the involved side, and a slight deviation of the labial sulcus, all also mentioned in the literature. Facial paralysis is rarely reported, as it is found only in case of very large tumors^1^, ^2^, ^9^.

Otoneurological tests indicated right ear areflexia, the most common finding in patients submitted to electronystagmography^1^, ^2^, ^9^.

Brainstem evoked response audiometry could not be performed, as the patient is in custody. However, we would expect to find an interval greater than 2.3ms between waves I and III; increased interval between waves I and V above 4.4ms; interaural difference on wave V greater than 0.4ms; and absent wave I.

## CONCLUSION

Vestibular schwannomas may be present and introduce only vestibular disorders such as instability during gait, either associated or not with unilateral tinnitus, in patients without complaints of hypoacusis. We should pay attention to such fact when diagnosing patients for vestibular schwannoma.
